# Effect of once versus twice intracoronary injection of allogeneic-derived mesenchymal stromal cells after acute myocardial infarction: BOOSTER-TAHA7 randomized clinical trial

**DOI:** 10.1186/s13287-023-03495-1

**Published:** 2023-09-23

**Authors:** Armin Attar, Mohsen Farjoud Kouhanjani, Kamran Hessami, Massoud Vosough, Javad Kojuri, Mani Ramzi, Seyed Ali Hosseini, Marjan Faghih, Ahmad Monabati

**Affiliations:** 1 Department of Cardiovascular Medicine, TAHA Clinical Trial Group, School of Medicine, Shiraz University of Medical Sciences, Zand Street, Shiraz, 71344-1864 Iran; 2 School of Medicine, Shiraz University of Medical Sciences, Shiraz, Iran; 3 Department of Regenerative Medicine, Cell Science Research Center, Royan Institute for Stem Cell Biology and Technology, ACECR, Tehran, Iran; 4 Department of Cardiovascular Medicine, Shiraz University of Medical Sciences, Shiraz, Iran; 5 Hematopathology and Molecular Pathology Service, Department of Pathology, Hematology Research Center, Shiraz University of Medical Sciences, Shiraz, 71344-1864 Iran; 6 Department of Biostatistics, School of Medicine, Arak University of Medical Sciences, Arak, Iran; 7 Department of Pathology, Shiraz University of Medical Sciences, Shiraz, Iran

**Keywords:** Myocardial infarction, Mesenchymal stromal cell, Intracoronary injection, Ventricular function, Stroke volume

## Abstract

**Background:**

Mesenchymal stromal cell (MSC) transplantation can improve the left ventricular ejection fraction (LVEF) after an acute myocardial infarction (AMI). Transplanted MSCs exert a paracrine effect, which might be augmented if repeated doses are administered. This study aimed to compare the effects of single versus double transplantation of Wharton’s jelly MSCs (WJ-MSCs) on LVEF post-AMI.

**Methods:**

We conducted a single-blind, randomized, multicenter trial. After 3–7 days of an AMI treated successfully by primary PCI, 70 patients younger than 65 with LVEF < 40% on baseline echocardiography were randomized to receive conventional care, a single intracoronary infusion of WJ-MSCs, or a repeated infusion 10 days later. The primary endpoint was the 6-month LVEF improvement as per cardiac magnetic resonance (CMR) imaging.

**Results:**

The mean baseline EF measured by CMR was similar (~ 40%) in all three groups. By the end of the trial, while all patients experienced a rise in EF, the most significant change was seen in the repeated intervention group. Compared to the control group (*n* = 25), single MSC transplantation (*n* = 20) improved the EF by 4.54 ± 2%, and repeated intervention (*n* = 20) did so by 7.45 ± 2% when measured by CMR imaging (*P* < 0.001); when evaluated by echocardiography, these values were 6.71 ± 2.4 and 10.71 ± 2.5%, respectively (*P* < 0.001).

**Conclusions:**

Intracoronary transplantation of WJ-MSCs 3–7 days after AMI in selected patients significantly improves LVEF, with the infusion of a booster dose 10 days later augmenting this effect.

*Trial registration*: Trial registration: Iranian Registry of Clinical Trials, IRCT20201116049408N1. Retrospectively Registered 20 Nov. 2020, https://en.irct.ir/trial/52357

**Supplementary Information:**

The online version contains supplementary material available at 10.1186/s13287-023-03495-1.

## Background

Coronary artery disease can lead to a myocardial infarction (MI), representing one of the leading causes of death globally [1]. Although the recent decades have seen a decrease in post-MI mortality, this has been coupled with an increase in heart failure (HF) [2]. Roughly 14–36% of patients hospitalized due to an acute myocardial infarction (AMI) develop HF [3]. This chronic condition burdens the health system, accounting for annual healthcare costs of roughly 40 billion USD in America [4]. Furthermore, mortality and morbidity rates of post-MI HF remain high despite the therapies available [5–7], with current treatments not failing to regenerate the damaged cardiac tissues. Hence, the need for novel strategies is strongly felt [8], with stem-cell-based therapies presenting as a promising option [9].

Beginning in the late twentieth century, the possibility of regenerating cardiac tissue using stem cells was explored in pre-clinical studies [10–13] before swiftly transitioning to the clinical phase [14, 15]. One safe and highly available stem cell population for such applications is mesenchymal stromal cells (MSCs), which can be isolated from the bone marrow, heart, Wharton’s jelly, and adipose tissue [16, 17]. These cells are easy to isolate and grow ex vivo and have excellent characteristics for in vivo usage [18]. Furthermore, allogeneic and autologous MSCs reportedly offer equal safety and efficacy [19], with one trial on cardiomyopathy patients, indicating that MSCs have twice the efficacy of bone marrow-derived mononuclear cells (BM-MNCs) [20]. Accordingly, MSCs appear to be the optimal option for cardiac stem-cell-based therapies.

Studies on the use of MSCs in AMI have provided promising yet controversial results. The most extensive trial was conducted by Gao et al. on 116 patients, reporting that these stem cells augmented the LVEF by almost 5% [21]. A related meta-analysis noted a 3.84% rise in LVEF [22], with the treatment benefiting relatively younger patients with a reduced LVEF the most. It appears that transplanted MSCs exert an indirect paracrine effect, and direct differentiation to cardiomyocytes does not occur [23]. Hence, we hypothesized that the paracrine effect of MSCs would be augmented if repeated doses were transplanted. Accordingly, we conducted a randomized clinical trial to compare the efficacy of single vs. double injection of MSCs in treating post-AMI heart failure.

## Methods

### Study design

**Fig. 1:**
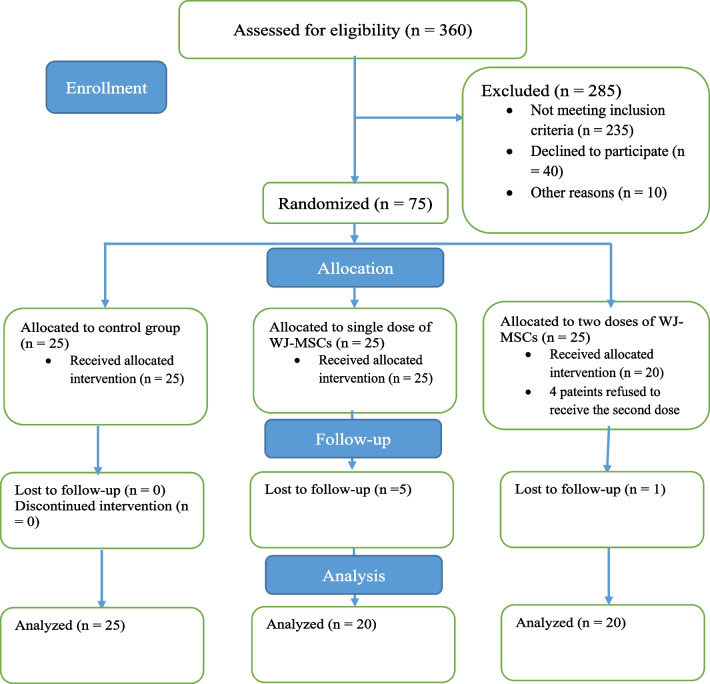
CONSORT flow diagram of the study

We conducted a randomized, single-blind, multicenter phase II trial to determine the effects of one or two intracoronary infusions of umbilical cord Wharton’s jelly tissue-derived MSCs on post-AMI LVEF when added to standard management. The Ethics Committee of Shiraz University of Medical Sciences approved the study protocol (IR.SUMS.REC.1399.406), which is registered with the Iranian Registry of Clinical Trials (IRCT20201116049408N1). Written informed consent was obtained from all patients before participation once they were in stable condition and recovered from the influence of sedatives. An independent Data and Safety and Monitoring Board (DSMB) monitored patient safety during the study. In-depth details regarding the study protocol are available in a previous publication [24] (Fig. 1).

### Sample size determination

Based on the study objectives and related literature, the sample size for each group was formulated as $$n = \frac{{2s^{2} (z_{{1 - {\raise0.7ex\hbox{$\alpha $} \!\mathord{\left/ {\vphantom {\alpha 2}}\right.\kern-0pt} \!\lower0.7ex\hbox{$2$}}}} + z_{1 - \beta } )^{2} }}{{(\partial )^{2} }}$$. The parameters used included 5% error, 90% power, 3% difference between placebo and single injection group, and 3% between single and double injection groups at the end of the 6 months of follow-up, with a standard deviation of 1.3 and a ratio of 1:1:1. Considering the study length and need for repeated measurements, the formula $$n^{\prime} = n \times \frac{1}{1 - p}$$ was also used with a drop-out rate of 15%, resulting in a minimum of 16 participants in each group. Ultimately, to allow comparisons between all three groups, the formula $$n^{\prime} = n \times \sqrt k$$ revealed the need for at least 20 subjects in each group.

### Study participants

Seventy patients hospitalized at Al-Zahra, Nemazee, or Faghihi Hospital (Shiraz, Iran) following an anterior ST elevation myocardial infarction (STEMI) and treated with a successful primary percutaneous coronary intervention (PCI) were included in this study. Inclusion criteria consisted of: 1—patients were 20–60 years old, 2—had their MI 3–7 days earlier, 3—had an echocardiographic LVEF < 40%. Exclusion criteria were: 1—prior history of an anterior MI, 2—regional wall motion abnormalities in the non-infarct region, 3—history of coronary artery bypass graft (CABG) surgery, 4—significant valve disease (stenosis/regurgitation grade ≥ 2), 5—alternative etiology of LV dysfunction (non-ischemic cardiomyopathy, 6—anthracycline therapy, 7—regular ethanol abuse > 6 oz. ethanol/day), 8—poor echocardiography window, 9—active syphilis, 10—hepatitis B, hepatitis C, HIV or HTLV-1, 11—terminal disease or cancer, 12—history of a bone marrow transplant, or an autoimmune disease, 13—being pregnant.

### Randomization and intervention

Patients were randomized using a web-based randomization service (https://www.sealedenvelope.com/simple-randomiser/v1/lists) into three groups with a 1:1:1 allocation ratio via permuted block randomization with a block size of 6. The outcome assessors were kept blinded to the group allocations. Following randomization, 30 patients received standard guideline-directed medical care, 20 received standard care plus a single intracoronary infusion of 10^7^ hWJ-MSCs 3–7 days after the AMI and 20 received standard care, the initial infusion, plus a repeated infusion after 10 days.

According to previous clinical and animal model studies, among the various sources of MSCs including bone marrow, adipose tissue, cord blood, Wharton’s jelly, etc., WJ-MCS were more efficacious in myocardial infarction and showed lower immunogenicity, higher immunomodulatory effect, smaller cell sizes and higher replication rate [25]. Accordingly, we used MSCs as the stem cell source for this study.

The reason for transplanting 10 million cells goes back to the animal studies showing that a higher number of MSCs may cause microvascular obstruction [26, 27] and also results from clinical trials showing that higher numbers would cause lower improvements in LVEF [22].

On each day of infusion, fresh cGMP-certified clinical-grade hWJ-MSCs (Cell Tech Pharmed Co. Ltd., Tehran, Iran) were transported to the catheterization laboratory in 0.9% normal saline. Cells were provided at the same day that procedure was planned to be performed from a cell bank. Qualification was performed according to GMP grade qualification protocols, and viability was assessed with methylene blue before transplantation. More details are provided on the published protocol of study [24].

The patients were returned to the catheterization laboratory and received heparin if their activating clotting time was below 200 s. A therapeutic 6-Fr guiding catheter was inserted into the left main artery, and 200 μg of nitroglycerin was infused using the guiding catheter. Coronary angiography was done, and TIMI flow was documented. A 0.014-inch soft-tipped guidewire wire was entered into LAD distal to the stent. Vessel occlusion was achieved by inserting and inflating an over-the-wire balloon within the stented area. After removing the guidewire, an infusion syringe was connected to the infusion catheter. The micro-infusion of MSCs was initiated at 2.5 ml/min, and the cells were infused in three equal portions. Prior to the infusion of each portion, total arterial occlusion was confirmed through dye injection, and TIMI coronary flow was assessed. Intracoronary cell infusion complications were averted by the use of low balloon inflation pressure [2–4 bar] and divided infusion portions.

### Safety monitoring

For monitoring the safety of treatment, a cardiologist visited the patients daily during hospitalization. A physical examination was conducted, and vital signs were noted. The patients were monitored for indicators of arrhythmia, pulmonary embolism, and coronary artery injury. Baseline tests including fasting blood sugar (FBS), complete cell blood count (CBC), urea and electrolytes, liver function tests (LFTs), creatine kinase, and cardiac troponin T and C reactive protein were also requested. A standard 12-lead electrocardiogram (ECG) and echocardiography were also taken. Safety measure was monitored using a standard questionnaire presented in Additional file 1. Subjects were examined for adverse events for 6 months after treatment. All adverse events graded 2 or higher in severity using the NCI Common Terminology Criteria for Adverse Events (version 4.0) were submitted by the clinical centers to the Data Coordinating Center safety team at UTHealth (Houston, Texas) for records review and sponsor assessment.

### Outcome measures and follow-up

A cardiologist visited the patients daily during hospitalization. A physical examination was conducted, and vital signs were noted. The patients were monitored for indicators of arrhythmia, pulmonary embolism, and coronary artery injury. Baseline tests including fasting blood sugar (FBS), complete cell blood count (CBC), urea and electrolytes, liver function tests (LFTs), creatine kinase, and cardiac troponin T and C reactive protein were also requested. A standard 12-lead electrocardiogram (ECG) was also taken. LV function was first evaluated via echocardiography, with the EF being calculated using the motion score and Simpson’s rule. The global longitudinal strain (GLS) was also measured with automated formulas in standard views. Cardiac magnetic resonance (CMR) imaging was done 3 days after primary PCI. Cine-CMR evaluated ventricular function and volume, delayed enhancement (DE)-CMR determined microvascular obstruction and infarct size, and T2 imaging assessed myocardial salvage and infarct size. Myocardial nulling was optimized according to the inversion time. Scar and edema volumes were manually traced with endocardial and epicardial contours after the semi-automated selection of the normal remote myocardium per slice. Images were assessed by an expert operator who was blinded to the study group allocations.

All patients were discharged with a beta-blocker, angiotensin-converting enzyme (ACE) inhibitor, aldosterone antagonist, aspirin, ticagrelor, statin, nitrates (as needed), and a cardiac rehabilitation program. The physical examination, blood tests, and ECG were repeated on day 10 and after 3 and 6 months. Echocardiography and CMR were repeated after 6 months. Follow-up visits were at Imam Reza Clinic (Shiraz, Iran) or the outpatient cardiology clinic of Al-Zahra Heart Hospital (Shiraz, Iran).

The primary outcomes were both safety and the LVEF improvement after 6 months. Secondary outcomes included the 6-month CMR-recorded change in the infarct area and echographic changes in LV function, left ventricular mass (LVM), left ventricular end-diastolic volume (LVEDV), left ventricular end-systolic volume (LVESD), and GLS.

### Statistical analysis

An independent, blinded expert evaluated and judged all measurements and excluded those of inadequate quality from the analysis. Then, the data were analyzed with an intention-to-treat analysis. In line with the literature, we considered a 6-month 3% improvement in EF as significant [22]. Baseline data were compared between the study arms and reported as mean and standard deviation of continuous and as frequencies and percentages if categorical. Changes in study outcomes were assessed between groups using one-way analysis of variance (ANOVA) coupled with Tukey’s post hoc test. Only patients who completed the study were included in the final analysis. Within-group changes were assessed via the paired t*-*test. The estimated treatment effect was reported with its 95% confidence interval (CI). Two-sided *P* values were obtained. Major adverse cardiac events (MACEs) and serious adverse events (SAEs) were compared between the three groups, with Kaplan–Meier curves plotted to show these events' patterns during follow-up. The Cox’s proportional hazards model was used to determine the hazard ratios with 95% CIs. The group allocation codes were kept blinded until after the analysis.

## Results

The baseline characteristics of patients are summarized in Table 1. As evident, the study groups were comparable in most baseline parameters, with a mean age of 55.46 ± 7.14 years. A male predominance was seen in all groups. Notably, most patients were smokers, and roughly a quarter had hypertension (Table 1).

**Table 1:** Baseline characteristics of patients, mean ± SD or *n* (%)

Characteristic	Total (*n* = 65)	Control (*n* = 25)	Intervention (*n* = 20)	Repeated intervention (*n* = 20)
Age, years	55.46 ± 7.14	56.53 ± 7.95	53.25 ± 5.05	56.05 ± 7.48
WBC/μl	9220 ± 2365.24	9253.33 ± 2550.69	9350 ± 2029.39	9040 ± 2492.81
Hb, g/dl	13.77 ± 1.89	13.61 ± 1.63	14.08 ± 2.21	13.71 ± 1.99
Platelets/μl	207,257.14 ± 70,193.25	224,866.67 ± 83,992.5	187,250 ± 30,654.05	200,850 ± 72,329.13
BUN, mg/dl	16.71 ± 4.68	17.33 ± 4.96	16 ± 4.04	16.5 ± 4.93
Cr, mg/dl	1.41 ± 2.13	1.83 ± 3.04	1 ± 0.25	1.04 ± 0.21
Na	138.91 ± 3.47	139.33 ± 3.63	138.5 ± 3.44	138.7 ± 3.36
K	4.08 ± 0.32	4.08 ± 0.39	4.1 ± 0.16	4.08 ± 0.33
SBP	128.36 ± 16.67	130 ± 19.63	125 ± 8.89	129.25 ± 18.01
DBP	80.29 ± 10.17	81.67 ± 12.16	77.5 ± 4.44	81 ± 10.86
Gender (male)	58 (82.9%)	22 (73.3%)	20 (100%)	16 (80%)
DM (yes)	8 (11.4%)	6 (20%)	0 (0%)	2 (10%)
HTN (yes)	18 (25.7%)	8 (26.7%)	5 (25%)	5 (25%)
Smoking (yes)	42 (60%)	14 (46.7%)	15 (75%)	13 (65%)
HLP (yes)	8 (11.4%)	6 (20%)	0 (0%)	2 (10%)

*WBC* white blood cells, *Hb* hemoglobin, *BUN* blood urea nitrogen, *Cr* creatinine, *Na* sodium, *K* potassium, *SBP* systolic blood pressure, *DBP* diastolic blood pressure, *DM* diabetes mellitus, *HTN* hypertension, *HLP* hyperlipidemia

The mean baseline EF measured by CMR was roughly 40% in all three groups (*P* = 0.392), as evident in Table 2. The final EF was significantly higher in the repeated intervention group relative to the control group (*P* = 0.003). By the end of the trial, while all patients experienced a rise in EF, the most significant change was in the repeated intervention group, where the EF increased on average by 10.24%—a 7.55% greater increase than that seen in the control group (*P* = 0.001) (Table 2).

**Table 2:** Changes in study outcome measures according to cardiac magnetic resonance imaging, mean ± SD and median (Q3–Q1)

	Control (*n* = 25)	Intervention (*n* = 20)	Repeated intervention (*n* = 20)	*P* value
EF baseline	40.33 ± 7.38 41 (47–35)	40.5 ± 6.35 42.5 (46–33)	39.5 ± 5.75 41.5 (45–32)	0.392^a^
EF final	43.03 ± 7.5 43.6 (47.2–38.3)	47.74 ± 9.87 51.2 (55.6–36.5)	49.74 ± 10.12 53.2 (57.6–38.5)	0.003^a^
EF change	2.69 ± 8.96 4.7 (9.7–(− 1.6))	7.24 ± 4.78 8.1 (11.9–1.7)	10.24 ± 5.87 11.1 (14.9–4.7)	0.001^a^
LVEDV baseline	184.5 ± 13.28 184.5 (196–173)	177 ± 30.57 177 (206–148)	181 ± 31.27 181 (210–152)	0.274^a^
LVEDV final	231 ± 12.73 231 (.–222)	140.8 ± 9.26 142 (149.5–131.5)	137 ± 5.83 137 (142–132)	0.53^b^
LVEDV change	58 ± 12.73 58 (.–49)	− 7.2 ± 9.26 − 6 (1.5–(− 16.51))	− 15 ± 5.83 − 15 (− 10–(− 20))	0.175^b^
LVESV baseline	113.93 ± 029 113.9 (114.2–113.7)	95.58 ± 16.51 95.6 (111.2–79.9)	99.55 ± 16.81 99.6 (115.5–83.6)	0.023^a^
LVESV final	133.98 ± 7.4 133.9 (.–128.8)	60.54 ± 3.98 61.1 (64.3–56.6)	56.17 ± 2.39 56.2 (58.2–54.1)	0.076^b^
LVESV change	19.8 ± 7.38 19.8 (. –14.6)	− 19.38 ± 3.9 − 18.9 (− 15.6–(− 23.4)	− 27.43 ± 2.4 − 27.4 (− 25.4–(−29.5))	0.016^b^

^a^Kruskal–Wallis test

^b^Mann–Whitney test

*EF* ejection fraction, *LVEDV* left ventricular end-diastolic volume, *LVESV* left ventricular end-systolic volume

Table 3 compares the diastolic function parameters measured by echocardiography between the groups. Pairwise comparisons revealed that the control group had a significantly lower E final than the intervention (*P* = 0.006) and repeated intervention (*P* = 0.013) groups. Regarding the e’ baseline, the single intervention group had a higher value than the control (*P* = 0.002) and repeated intervention (*P* = 0.008) groups. The control group had a significantly lower e’ final relative to the repeated (*P* = 0.019) and single intervention (*P* = 0.002) groups. The baseline E/e’ was lower in the single intervention group than control (*P* = 0.001) and repeated intervention (*P* = 0.004) groups. This is while the control group had a significantly higher final E/e’ than the intervention (*P* = 0.005) and repeated intervention groups (*P* = 0.014) (Table 3).

**Table 3:** Diastolic function parameters compared between the study groups, mean ± SD; median (Q3–Q1)

	Control (*n* = 25)	Intervention (*n* = 20)	Repeated intervention (*n* = 20)	*P* value
E baseline	0.63 ± 0.24 0.56 (0.64–0.46)	0.57 ± 0.19 0.53 (0.82–0.37)	0.64 ± 0.24 0.58 (0.63–0.46)	0.511
E Final	0.73 ± 0.21 0.62 (0.91–0.57)	0.58 ± 0.19 0.54 (0.8–0.41)	0.58 ± 0.17 0.56 (0.61–0.51)	0.002
A baseline	0.64 ± 0.15 0.66 (0.77–0.57)	0.64 ± 0.16 0.56 (0.85–0.51)	0.68 ± 0.14 0.75 (0.77–0.58)	0.649
A Final	0.68 ± 0.2 0.66 (0.72–0.59)	0.62 ± 0.08 0.66 (0.67–0.53)	0.62 ± 0.07 0.65 (0.67–0.57)	0.386
*e*′ baseline	0.05 ± 0.01 0.05 (0.06–0.04)	0.08 ± 0.03 0.08 (0.11–0.05)	0.05 ± 0.01 0.05 (0.06–0.04)	0.001
*e*′ final	0.6 ± 1.89 0.06 (0.08–0.05)	1.81 ± 3.08 0.08 (5.27–0.07)	1.53 ± 2.9 0.08 (0.09–0.06)	0.001
EA-ratio baseline	0.95 ± 0.31 0.8 (1.1–0.73)	0.88 ± 0.17 0.96 (1.04–0.66)	0.93 ± 0.33 0.78 (1.1–0.72)	0.779
E/A-ratio final	1.19 ± 0.67 0.97 (1.2–0.88)	0.96 ± 0.31 0.95 (1.03–0.62)	0.95 ± 0.29 0.97 (1.1–0.76)	0.6
*E*/*e*′-ratio baseline	13.15 ± 6.58 11.7 (15–7.7)	7.56 ± 2.53 7.5 (10.6–4.6)	12.65 ± 5.91 11.9 (15–7.6)	< 0.001
*E*/*e*′-ratio final	12.04 ± 6.34 11.3 (17.5–8.2)	5.95 ± 4.27 6.36 (10.4–1.1)	6.33 ± 4.2 8.5 (10.4–3.2)	0.002

The mean baseline EF, as measured by echocardiography, was around 34%, with the repeated intervention group having a significantly lower baseline EF than the control group (*P* = 0.013) (Table 4). By the end of the trial, while the echocardiographic EF increased in all groups, this increase was more than twice greater in the repeated intervention group (19.25 ± 8.35%) relative to the control group (8.53 ± 9.39%) (*P* < 0.001). Also, the LVESD saw a 6.62 ± 7.9 mm increase in the control group compared with a 4.65 ± 14.55 mm decrease in the repeated intervention group (*P* = 0.008). While a decrement in GLS was seen in all groups, the single intervention (*P* = 0.015) and repeated intervention (*P* < 0.001) groups had roughly two and three times greater decrements in GLS than the control group, respectively (Table 4).

**Table 4:** Changes in study outcome measures according to echocardiography, mean ± SD and median (Q3–Q1)

	Control (*n* = 25)	Single intervention (*n* = 20)	Repeated intervention (*n* = 20)	*P* value
EF baseline	35.73 ± 3.59 36 (38–32)	34.75 ± 1.97 34 (37–33.3)	33.85 ± 1.67^a^ 33 (36–32.3)	0.066
EF final	44.27 ± 8.65 43 (49–39)	50 ± 10.21 49 (61–40)	53 ± 12.11^a^ 52 (64–43)	0.006
EF change	8.53 ± 9.39 8 (15–3)	15.25 ± 8.35 15 (24–6.8)	19.25 ± 9.12^a^ 19 (28–10.8)	< 0.001
LVEDD baseline	50.92 ± 6.16 51 (56–44.8)	54.5 ± 5.87 55 (60.5–48)	55.4 ± 7.65^a^ 56.5 (62.5–49)	0.04
LVEDD final	55.8 ± 5.23 57 (59–51)	57.25 ± 11.27 52.5 (70.3–49)	54.25 ± 10.67^a^ 49.5 (67.3–46)	0.031
LVEDD change	5.46 ± 7.37 6 (9.5–(− 0.3))	2.75 ± 10.96 3.5 (14–(− 9.3))	− 1.15 ± 11.59 0 (12–(− 14))	0.099
LVESD baseline	36.92 ± 6.58 37 (41.3–31.5)	36.75 ± 6.7 37.5 (43.5–29.3)	39.65 ± 6.96 43 (45–31.5)	0.317
LVESD final	42.53 ± 8.32 38 (47–37)	39 ± 17.74 34 (60–23)	35 ± 16.94 30 (56–19)	0.083
LVESD change	6.62 ± 7.9 2 (10–1.8)	2.25 ± 14.83 1.5 (18.8–(− 13.5))	− 4.65 ± 14.55^a^ − 11 (12.5–(− 19.3))	0.01
LVEDV baseline	98.08 ± 23.55 101.5 (116.3–78.5)	118.25 ± 22.61 123.5 (139–92.3)	123.25 ± 21.91^a^ 128.5 (144–128.5)	0.002
LVEDV final	101.33 ± 23.94 103 (125–78)	121.75 ± 50.33 114 (177.3–74)	116.75 ± 48.93 109 (172.3–69)	0.19
LVEDV change	4.58 ± 21.15 0.5 (28.8–(− 15.5))	3.5 ± 3.23 1 (51.3–(− 41.75))	− 6.5 ± 4.32 − 9 (41.3–(− 51.8))	0.39
LVESV baseline	63.25 ± 14.54 65.5 (73.5–49.7)	77.31 ± 15.91 79.4 (92.8–59.8)	81.81 ± 16.18^a^ 83.8 (97.6–64)	0.003
LVESV final	56.8 ± 16.49 62.1 (69–40.3)	65.59 ± 38.28 58.1 (108.2–30.4)	59.59 ± 36.28 52.3 (100–26.4)	0.392
LVESV change	− 5.68 ± 16.62 − 5.1 (4.6–(− 20.1))	− 11.72 ± 5.18 − 16.4 (24–(− 42.7))	− 22.22 ± 9.26 − 26.7 (11.2–(− 51.11))	0.015
GLS baseline	− 9.84 ± 2.6 − 9.5 (− 8.6–(− 11))	− 9.28 ± 2.34 − 10.1 (− 6.5–(− 11.2))	− 8.28 ± 1.95 − 9.1 (− 5.5–(10.2))	0.092
GLS final	− 14.13 ± 2.77 − 14.2 (− 13–(− 16.5))	− 16.05 ± 3.85 − 16.5 (− 11.7–(− 19.9))	− 18.05 ± 3.75^a^ − 18.6 (− 13.7–(− 21.9))	0.007
GLS change	− 3.92 ± 2.35 − 3.7 (− 2.9–(− 4.81))	− 6.77 ± 3.05^a^ − 7.4 (− 3.3–(− 9.6))	− 9.77 ± 3.65^a^ − 10.4 (− .3–(− 12.6))	< 0.001

^a^Significant difference with the control group on the post hoc test

^b^Significant difference with the single intervention group on the post hoc test

*EF* ejection fraction, *LVEDD* left ventricular end-diastolic diameter, *LVESD* left ventricular end-systolic diameter, *LVEDV* left ventricular end-diastolic volume, *LVESV* left ventricular end-systolic volume, *GLS* global longitudinal strain

Table 5 displays the measurements of infarct size, indicating that the reduction in the amount of scared myocardium was more pronounced in the repeated intervention group.

**Table 5:** Infarct size (mass) measured by cardiac magnetic resonance imaging

	Mean	Std. deviation	*P* value
Baseline infarct size			
Control	22.80	9.385	0.162
Intervention	17.74	4.610	
Repeated intervention	27.80	15.556	
Final infarct size			
Control	13.00	2.828	0.175
Intervention	6.98	1.597	
Repeated intervention	8.00	1.000	
Infarct size change			
Control	− 9.8	2.0	0.03
Intervention	− 12.1	5.81	
Repeated intervention	− 18.47	9.77	

Unit of measurement is grams

Regarding safety outcomes, the intracoronary infusion of MSCs resulted in no adverse effects such as cardiac arrhythmias, re-flow phenomenon, or TIMI flow decrease in the intervention or repeated intervention groups. Furthermore, no intracardiac tumorigenic effects were noticed.

## Discussion

The present study revealed that two post-AMI intracoronary MSC infusions could significantly improve LVEF on both CMR imaging and echocardiography. Compared to the control group, single MSC transplantation improved the EF by 4.54 ± 2%, and repeated intervention did so by 7.45 ± 2% when measured by CMR imaging (*P* < 0.001); when evaluated by echocardiography, these values were 6.71 ± 2.4 and 10.71 ± 2.5%, respectively (*P* < 0.001).

Currently, post-AMI therapeutic measures seek to avert cardiac remodeling and myocyte loss [5]. This is while stem-cell-based therapies go one step further and attempt to reverse the damage through the provision of fresh, functional cells [21]. While early studies on this topic were not very promising [9], later meta-analyses showed that this therapy might be effective in certain populations [22, 24, 28–32]. Hence, in the present study, we selected patients below 65 with an echocardiographic LVEF < 40%, yielding promising results.

One issue with cardiac stem cell therapy is the type of cell used. A Cochrane meta-analysis showed that treating AMI patients with a reduced LVEF with BM-MNCs effectively increases LVEF, yielding survival and functional benefits in patients below 55 with an EF below 37% [33]. On the other hand, two more recent meta-analyses found that while post-AMI therapy with BM-MNCs can decrease hospitalization for CHF and re-infarction, their effect on all-cause mortality and stroke rate is questionable [29, 30]. Another cell type is MSCs, which were about twice as effective as BM-MNCs in the TAC-HFT trial [20]. Meta-analyses indicate that MSCs can improve LVEF by 3.84% [22], compared with 2.72% in BM-MNCs [33]. In the only meta-analysis (36 trials; 2,489 patients) to directly compare MSCs with BM-MNCs, the former performed superiorly than the latter (3.67% vs. 2.13%), particularly when therapy was delivered within the first 10 days of an AMI (5.65% vs. 3.07%) [28]. Furthermore, the POSEIDON trial indicated that allogeneic MSCs are as safe and effective as autologous MSCs [34]. Hence, MSCs are more accessible and effective in regenerative cardiology than BM-MNCs, and our preference for MSCs in the present trial is vehemently justified.

The route of stem cell injection has also been a subject of study. In an animal study, Gong et al. showed that a repeated intravenous dose of human umbilical cord-derived MSCs had a superior therapeutic effect than single-dose treatment in improving the LV function of rats with dilated cardiomyopathy [35]. In a groundbreaking phase 1 trial, Hsiao et al. found that combined intracoronary and intravenous (2 days apart) umbilical cord-derived MSCs appeared to be safe, feasible, and effective (9.80 ± 7.56% rise in EF after 12 months), though confirmatory phase 2 studies are needed [36]. On the other hand, we delivered the stem cells via an intracoronary micro-infusion, which a meta-analysis revealed to have similar efficacy as a transendocardial injection [22].

The timing of delivery of stem cells for following an AMI is a crucial therapeutic parameter. Numerous trials [37–39] and meta-analyses [40, 41] have examined this matter on BM-MNCs, revealing an optimal transplantation time of 3–7 days after an AMI. If done sooner, the transplanted cells might be lost, considering the myocardium’s highly inflammatory state; if delayed, it may be less effective due to myocyte loss and fibrosis. While this issue has not been directly evaluated in trials on MSCs, meta-analyses indicate that the first dose would be more effective if transplanted within 7 to 10 days of an AMI [22, 28].

Regarding the number of MSCs used, the current understanding is that the optimal number is 10^7^ cells, with fewer or more cells not altering the outcomes [22]. Studies on pigs and sheep indicate that transplanting excessive MSCs into the heart can induce microvascular obstruction and myocardial injury [26, 27]. Hence, we used 10^7^ cells.

The literature does not support the theory of differentiation into cardiomyocytes as the mechanisms behind the therapeutic effects of stem cells on post-AMI heart, as the number of cells required to account for such effects is immense [42]. Differentiation into vessels is also possible [43], where vasculogenesis would rescue hypoxic cardiomyocytes. However, this is probably not the main mechanism, as patients in all related trials (including the present study) had undergone revascularization or had received thrombolytics [22]. The paracrine effect theory represents the current paradigm, where paracrine cytokine signaling by transplanted cells affects neighboring cells in the recipient’s heart [23]. Hence, we hypothesized that a repeated dose would induce a better therapeutic effect, which was confirmed by our findings. Similarly, Yao et al. found that a repeated dose of BM-MNCs was more effective than a single dose, though their second dose was delivered after 3 months [44]. In the rat study by Gong et al., the repeated intravenous administration of MSCs reduced myocardial inflammation while upregulating indoleamine 2,3-dioxygenase, which offers anti-inflammatory properties [35].

This study had some limitations. Firstly, while we originally planned to conduct a sham procedure as a placebo treatment for the control group [24], this did not receive ethical clearance from our institute. Hence, the control group was restricted to standard guideline-directed medical therapy and cardiac rehabilitation after the successful primary PCI. Secondly, the duration of follow-up was 6 months, so the long-term effects could not be established. In addition, the patients could not be blinded regarding the group to which they were randomly allocated. Finally, we failed to assess the liquid levels of inflammatory markers before and after stem cell transplantation. Nonetheless, our study had some major strengths, including its robust protocol, use of both CMR and echocardiographic imaging, and blinding of the outcome assessors.

## Conclusion

Intracoronary transplantation of WJ-MSCs 3–7 days after AMI in patients younger than 65 with a baseline LVEF below 40% significantly improves LVEF, with the infusion of a booster dose 10 days later augmenting this effect.

### Supplementary Information

**Additional file 1.** Treatment toxicity.

## Data Availability

Data supporting the findings of this study are available from the corresponding author upon reasonable request.

## Abbreviations

MSCs: Mesenchymal stromal cells
LVEF: Left ventricular ejection fraction
AMI: Acute myocardial infarction
WJ-MSCs: Wharton’s jelly mesenchymal stromal cells
MI: Myocardial infarction
BM: Bone marrow
HF: Heart failure
BM-MNCs: Bone marrow-derived mononuclear cells
EF: Ejection fraction
CMR: Cardiac magnetic resonance
GLS: Global longitudinal strain
DE: Delayed enhancement
MACE: Major adverse cardiac events
SAE: Serious adverse events
DSMB: Data and Safety and Monitoring Board
