# Correction: Effect of once versus twice intracoronary injection of allogeneic-derived mesenchymal stromal cells after acute myocardial infarction: BOOSTER-TAHA7 randomized clinical trial

**DOI:** 10.1186/s13287-024-03663-x

**Published:** 2024-02-14

**Authors:** Armin Attar, Mohsen Farjoud Kouhanjani, Kamran Hessami, Massoud Vosough, Javad Kojuri, Mani Ramzi, Seyed Ali Hosseini, Marjan Faghih, Ahmad Monabati

**Affiliations:** 1 Department of Cardiovascular Medicine, TAHA Clinical Trial Group, School of Medicine, Shiraz University of Medical Sciences, Zand Street, Shiraz, 71344-1864 Iran; 2 School of Medicine, Shiraz University of Medical Sciences, Shiraz, Iran; 3 Department of Regenerative Medicine, Cell Science Research Center, Royan Institute for Stem Cell Biology and Technology, ACECR, Tehran, Iran; 4 Department of Cardiovascular Medicine, Shiraz University of Medical Sciences, Shiraz, Iran; 5 Hematopathology and Molecular Pathology Service, Department of Pathology, Hematology Research Center, Shiraz University of Medical Sciences, Shiraz, 71344-1864 Iran; 6 Department of Biostatistics, School of Medicine, Arak University of Medical Sciences, Arak, Iran; 7 Department of Pathology, Shiraz University of Medical Sciences, Shiraz, Iran

**Correction: Stem Cell Research & Therapy (2023) 14:264** 10.1186/s13287-023-03495-1

In the original article, the authors identified that at the 3rd page of article in right column in the 12th line they had forgotten to add the name of the cell product used for the trial.

This line should be corrected as follows:

“On each day of infusion, fresh cGMP-certified clinical-grade hWJ-MSCs (Whartocell®, Cell Tech Pharmed Co. Ltd., Tehran, Iran) were transported to the catheterization laboratory in 0.9% normal saline."

