# Supplementary material for: Effect of once versus twice intracoronary injection of allogeneic-derived mesenchymal stromal cells after acute myocardial infarction: BOOSTER-TAHA7 randomized clinical trial
Source: Stem Cell Res Ther. 2023 Sep 23;14:264. doi: 10.1186/s13287-023-03495-1 (PMC10517503; doi:10.1186/s13287-023-03495-1)
Supplement: Supplementary file 1 — Additional file 1. Treatment toxicity. [file 13287_2023_3495_MOESM1_ESM.pdf]

فرم ارزیابی انواع عوارض گزارش شده

TREATMENT TOXICITY

1. Hematologic: 1 ❑ Yes 2 ❑ No

	Yes	No		Duration	Start date
**2.**	1 ❑	2 ❑	Leukopenia (WBC<2000)		
**3.**	1 ❑	2 ❑	Thrombocytopenia (Plt< 50000)		
**4.**	1 ❑	2 ❑	Anemia (Hb<8)		
**5.**	1 ❑	2 ❑	Granulocytopenia (PMN<900)		
**6.**	1 ❑	2 ❑	Hemorrhage (gross blood loss)		

7. Fever with drug (>40 °C) 1 ❑ Yes 2 ❑ No

8. Gastrointestinal: 1 ❑ Yes 2 ❑ No

	Yes	No		Duration	Start date
**9.**	1 ❑	2 ❑	Oral (ulcers requires liquid diet only or more sever)		
**10.**	1 ❑	2 ❑	Diarrhea (intolerable D.- requiring therapy and/or bloody D. and dehydration)		
**11.**	1 ❑	2 ❑	Nausea/vomiting (vomiting requiring therapy)		
**12.**	1 ❑	2 ❑	Bilirubin (>5×N[1])		
**13.**	1 ❑	2 ❑	SGPT/SGOT (>5×N[1])		
**14.**	1 ❑	2 ❑	Alk Pho (>5×N[1])		

(1) N= Upper limit of normal value of population.

15. Pulmonary (dyspnea at rest) 1 ❑ Yes 2 ❑ No

16. Cardiac: 1 ❑ Yes 2 ❑ No

	Yes	No		Duration	Start date
**17.**	1 ❑	2 ❑	Rhythm (multifocal PVC or ventricular tachycardia)		
**18.**	1 ❑	2 ❑	Function (symptomatic dysfunction responsive/non-responsive to therapy)		
**19.**	1 ❑	2 ❑	Pericarditis (tamponade required tap or surge[illegible]		

20. Renal: ₁ ☐ Yes ₂ ☐ No

(2) N= The institutional upper limit of

	Yes	No		Duration	Start date
21.	1 ☐	2 ☐	BUN, blood urea and Cr (>5×N(2))		
22.	1 ☐	2 ☐	Proteinuria (4+, >1.0 gm/100ml)		
23.	1 ☐	2 ☐	Hematuria (Gross+Clots)		

24. Neurotoxicity: ₁ ☐ Yes ₂ ☐ No

	Yes	No		Duration	Start date
25.	1 ☐	2 ☐	State of consciousness (somnolence> of waking hours or coma)		
26.	1 ☐	2 ☐	Peripheral (intolerable paresthesias ar marked motor loss or paralysis)		
27.	1 ☐	2 ☐	Constipation(3) (abdominal distention)		

(3) Constipation dose not include constipation resulting from

28. Allergic (bronchospasm requiring parenteral therapy, anaphylaxis)
₁ ☐ Yes ₂ ☐ No

29. Cutaneous (moist desquamation, ulceration, exfoliative dermatitis or necrosis requiring surgical intervention)
₁ ☐ Yes ₂ ☐ No

30. Hair (complete alopecia) ₁ ☐ Yes ₂ ☐ No

31. Infection major infection (required IV therapy) and/or hypotension
₁ ☐ Yes ₂ ☐ No

32. Pain(4) (sever or intractable) ₁ ☐ Yes ₂ ☐ No

(4) Pain: only treatment-related pain. The use of narcotics may be helpful in grading pain, depending [illegible]

33. Was there any site of Infection? 1 ❑ Yes 2 ❑ No

	Yes	No	
**34.**	1 ❑	2 ❑	Blood
**35.**	1 ❑	2 ❑	Skin
**36.**	1 ❑	2 ❑	Lung
**37.**	1 ❑	2 ❑	Urogenital
**38.**	1 ❑	2 ❑	GI
**39.**	1 ❑	2 ❑	Sinuses
**40.**	1 ❑	2 ❑	Other,

specify:

41. Any Discontinues of Drug? 1 ❑ Yes 2 ❑ No → ☐☐ ☐☐ ☐☐

Cuase:...

....

42. Date of discharge ☐☐ ☐☐ ☐☐

INVESTIGATOR NAME: .... DATE & SIGNATURE: ....
